# Spinocerebellar Ataxia Type 35 Caused by a New TGM6 Variant: Video Documentation of a German Family

**DOI:** 10.1002/mdc3.13717

**Published:** 2023-03-27

**Authors:** Fabian Maass, Ala Jamous, Saskia Biskup, Hanna Eisenberg, Zara D'Hedouville, Mathias Bähr, Christoph van Riesen

**Affiliations:** ^1^ Department of Neurology University Medical Center Göttingen Göttingen Germany; ^2^ Department of Neuroradiology University Medical Center Göttingen Göttingen Germany; ^3^ CeGaT, Center for Genomics and Transcriptomics Tübingen Germany; ^4^ Germany Center for Neurodegenerative Diseases (DZNE) Göttingen Germany

The Spinocerebellar ataxias represent a heterogeneous group of autosomal dominant transmitted neurodegenerative disorders. The number of causative genes is growing and up to now, more than 40 different SCAs have been described.^1^ Variants in the transglutaminase six gene (*TGM6*)—now known to cause ATX‐TGM6 (SCA35)—have been first described in 2010,^2^ but the clinical phenomenology has been only sparsely documented. A late onset slowly progressive cerebellar ataxia with gait problems, dysarthria, hand tremor, and mild abnormal eye movements has been reported to present the most common phenotype.^2^, ^3^ Less frequently, dystonia, myoclonus, and parkinsonism have been described. An episodic ataxia‐like phenotype has also been reported.^4^

There is some debate whether *TGM6* variants are causative for ATX‐TGM6 (SCA35), as benign or low penetrant variants might be misclassified as pathogenic and therefore, might also occur in patients with various neurologic diseases or normal controls.^5^, ^6^

TGM6 is neuronally expressed and catalyzes various post‐translational modifications in proteins and peptides,^7^ but its exact role in ATX‐TGM6 (SCA35) pathogenesis is still elusive. Antibodies against TGM6 have been discussed as a biomarker for gluten ataxia,^8^ so a converging mechanism for gluten ataxia and ATX‐TGM6 (SCA35) might be responsible for the evolving phenotype. Most affected patients were reported in in China and in Han‐Chinese descents in Taiwain.^3^ Here, we present to the best of our knowledge, the first description of a new pathogenic *TGM6* variant (c.1430_1431delins24; p.Gly477Valfs*15) in a German family, accompanied by ATX‐TGM6 (SCA35) manifestation.

A 72‐year‐old Caucasian woman (Video 1) was referred to our specialized outpatient clinic for movement disorders for genetic counseling. She reported progressive problems with coordination, first noticed 22 years ago. There was a slow progression leading to manifest gait problems ~10 years ago and assisted walking and recurrent falls within the last 2 years. Recently, speech and swallowing problems were noticed. She also reported slight subjective memory problems, as well as progressive urge urinary and bowel incontinence. Previous medical history revealed an unremarkable gene panel diagnostic for common causative genes in hereditary ataxias (ATXN1, ATXN2, ATXN3, CACNA1, ATXN7, ATXN8, ATXN10, PPP2R2B, and TBP). She reported a known coeliac disease, diagnosed by biopsy. Associated antibodies (anti‐deamidated gliadin, anti‐tissue transglutaminase/anti‐endomysial antibodies) could not be detected currently by us while the patient was on a gluten‐free diet. Her father also suffered from progressive speech and gait impairment of unknown etiology. He died at the age of 72 years.

**Video 1:** Neurological examination of patient 1 (72‐year‐old mother) revealing cerebellar symptoms including impaired smooth pursuit, slight upper limb ataxia, dysdiadochokinesia, cerebellar dysarthria, and a severe broad‐based ataxic gait.

Clinical examination revealed exophoria. She had undergone surgery to treat this condition 7 years earlier, but only had a transient reduction of double vision. There were cerebellar symptoms including impaired smooth pursuit, hypermetric saccades, slight limb ataxia, cerebellar dysarthria, slight dysdiadochokinesia, and a severe broad‐based ataxic gait with an additional sensory component, as revealed by a pathological Romberg's maneuver. In accordance, there was a loss of vibration sense, reflected by a 1/8 pallhypesthesia at the level of the malleoli. Scale for the Assessment and Rating of Ataxia (SARA) yielded 16.5/40 points. There were no extrapyramidal or pyramidal signs. Cognitive assessment using The Consortium to Establish a Registry for Alzheimer's Disease (CERAD) plus battery only revealed slight problems with word list learning, phonematic fluency and trail making. The Mini Mental State Examination (MMSE) yielded 29/30 points.

Magnetic resonance imaging (MRI) revealed cerebellar atrophy involving the cerebellar vermis and hemispheres. Furthermore, pontomesencephalic volume loss and thinning of the upper cerebellar peduncle could be demonstrated (Fig. 1A–C). Genetic sequencing revealed a heterozygous *TGM6* variant (c.1430_1431delins24; p.Gly477Valfs*15), consistent with an ATX‐TGM6 (SCA35) manifestation (technical details can be found in the Supporting Information).

**FIG 1:**
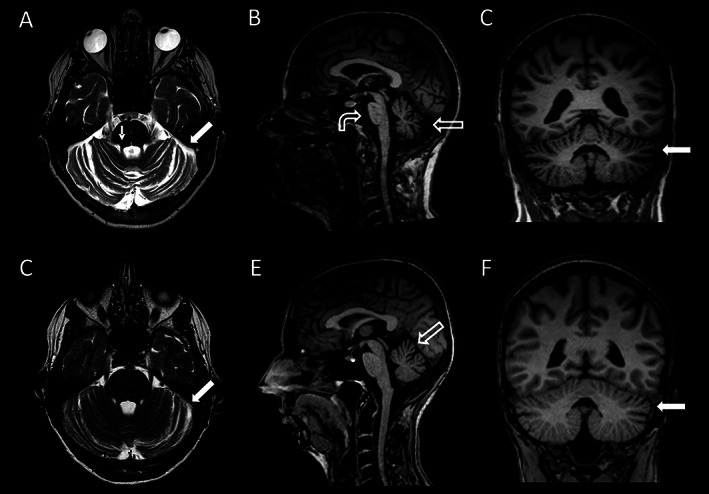
Fluid attenuated inversion recovery magnetic resonance imaging images in axial, coronal, and sagittal view. Mother (A–C), daughter (D–F). (A–C) showing advanced cerebellar atrophy involving the cerebellar vermis (open arrow in B), hemispheres (bold arrow in A and C), thinning of the upper cerebellar peduncle (thin arrow in A) and pontomesencephalic volume loss (curved arrow in B). (D–F) Showing accented slight left hemispheric thinning of the cerebellar folia (bold arrow in D and F) and predominantly upper vermal atrophy (open arrow in E).

The 52‐year‐old daughter (Video 2) was referred to our department, subsequently. She reported dizziness and impaired limb coordination, first recognized a few months ago. She also noticed a slight intermitting rest‐ and action tremor, first noticed 1 year ago. Clinical examination revealed slight limb ataxia of arms and legs, but no gait abnormalities (two points on SARA score). There were neither eye movement abnormalities nor speech problems. She reported slight problems with swallowing and first signs of possible urge incontinency. There was no involvement of the pyramidal system. Cognitive assessment applying CERAD plus battery revealed a slight impairment of sematic fluency. MMSE was normal (30/30 points). Serum transglutaminase antibodies were not detectable. Genetic sequencing revealed the same heterozygote *TGM6* variant, as described above. MRI revealed accented slight thinning of the cerebellar folia and predominantly upper vermal atrophy (Fig. 1D–F).

**Video 2:** Neurological examination of patient 2 (52‐year‐old daughter) revealing only slight limb ataxia and no gait abnormalities. There were neither eye movement abnormalities nor speech problems.

Here, we report a new *TGM6* variant (c.1430_1431delins24; p.Gly477Valfs*15), resulting in a reading frame shift, potentially leading to a non‐sense mediated mRNA decay. Other pathogenic *TGM6* frameshift variants leading to a ATX‐TGM6 (SCA35) phenotype have already been described.^9^, ^10^

SCA35 families are almost exclusively reported in families of Chinese origin and appearance in Europe has been investigated insufficiently. Recently, a first ATX‐TGM6 (SCA35) case has been reported in Italy.^11^ Another non‐Asian patient was reported from Puerto Rico, interestingly showing a clinical improvement on gluten‐free diet. Here, duodenal biopsy revealed normal mucosa without evidence of celiac disease.^12^ To the best of our knowledge, clinical signs or biopsy proven pathology hinting to celiac disease is not a common feature ATX‐TGM6 (SCA35).

Our case presentation is for the most part in‐line with the classical phenotype of ATX‐TGM6 (SCA35) as described in literature.^2^, ^3^ Interestingly, both of our patients presented with bladder and in the case of the mother, with bowel incontinency of unknown etiology, which might present an autonomous feature of this new variant. Erectile dysfunction as another possible symptom of autonomic dysfunction has been already reported.^11^ Brisk reflexes and pathological reflexes have been described frequently, but pyramidal involvement could not be detected in our patients with the new variant.^2^, ^3^

Our patients presented with normal MMSE scores and only slight cognitive abnormalities could be detected applying extensive neuropsychological testing (CERAD battery). In accordance, distinct cognitive decline has only been reported in subgroup of ATX‐TGM6 (SCA35) patients with a p.D510H variant.^3^

The described MRI features can also be found in our patients, mainly showing cerebellar and optional brainstem atrophy.^3^, ^11^

In conclusion, *TGM6* variants represent a potentially underestimated cause of ataxias in Europe and should be added to the typical spectrum of late onset hereditary ataxia like ATX‐ATXN3 (SCA3/MJD), ATX‐CACNA1A (SCA6), and ATX‐TBP (SCA17). Although cerebellar ataxia presents the central manifestation of ATX‐TGM6 (SCA35), different variants seem to modulate phenotype partially.

## Author Roles

(1) Research Project: A. Conception, B. Organization, C. Execution; (2) Statistical Analysis: A. Design, B. Execution, C. Review and Critique; (3) Manuscript Preparation: A. Writing of the First Draft, B. Review and Critique.

F.M.: 1A, 1B, 1C, 3A, 3B

A.J.: 1B, 1C, 3B

S.B.: 1B, 1C, 3B

H.E.: 1B, 1C, 3B

Z.H.: 1C, 3B

M.B.: 1B, 3B

C.v.R.: 1A, 1B, 1C, 3A, 3B

## Disclosures

**Ethical Compliance Statement:** The authors confirm that the approval of an institutional review board was not required for this work. We confirm that patient consent has been sought and allowed for this case and its publication. We confirm that we have read the Journal's position on issues involved in ethical publication and affirm that this work is consistent with those guidelines.

**Funding Sources and Conflicts of Interest:** No specific funding was received for this work. The authors declare that there are no conflicts of interest relevant to this work.

**Financial Disclosures for Previous 12 Months**: F.M. received speaker honoraria from AbbVie. C.v.R. received speaker honoraria from AbbVie and Zambon.

## Supporting information


**DATA S1.** Genetic testing methodology. Technical details concerning the genetic sequencing methods performed for the identification of the new TGM6 variant.
