# Supplementary material for: Spinocerebellar Ataxia Type 35 Caused by a New TGM6 Variant: Video Documentation of a German Family
Source: Mov Disord Clin Pract. 2023 Mar 27;10(6):1016–9. doi: 10.1002/mdc3.13717 (PMC10272897; doi:10.1002/mdc3.13717)
Supplement: Supplementary file 1 — DATA S1. Genetic testing methodology. Technical details concerning the genetic sequencing methods performed for the identification of the new TGM6 variant. [file MDC3-10-1016-s001.docx]

**Genetic testing methodology:**

Protein-coding regions, as well as flanking intronic regions and additional disease-relevant non-coding regions were enriched using in-solution hybridization technology and were sequenced using the Illumina NovaSeq6000 system. Illumina bcl2fastq2 was used to demultiplex sequencing reads. Adapter removal was performed with Skewer. The trimmed reads were mapped to the human reference genome (hg19) using the Burrows Wheeler Aligner. Reads mapping to more than one location with identical mapping score were discarded. Read duplicates that result from PCR amplification were removed. The remaining high-quality sequences were used to determine sequence variants (single nucleotide changes and small insertions/deletions). The variants were annotated on several internal and external databases. Rare variants were manually assessed before inclusion in the final report, classified and reported based on ACMG/ACGS-2020v4.01 guidelines.
